# Ebola Virus Activates IRE1α-Dependent *XBP1u* Splicing

**DOI:** 10.3390/v15010122

**Published:** 2022-12-30

**Authors:** Cornelius Rohde, Sebastian Pfeiffer, Sara Baumgart, Stephan Becker, Verena Krähling

**Affiliations:** 1 Institute of Virology, Philipps University Marburg, 35043 Marburg, Germany; 2 German Center for Infection Research (DZIF), Partner Site Gießen–Marburg–Langen, 35043 Marburg, Germany

**Keywords:** Ebola virus, Marburg virus, unfolded protein response, IRE1α, XBP1, ER stress, nucleoprotein, glycoprotein

## Abstract

Ebola (EBOV) and Marburg virus (MARV) are highly pathogenic filoviruses that influence cellular signaling according to their own needs. MARV has been shown to regulate the IRE1α-dependent unfolded protein response (UPR) to ensure optimal virus replication. It was not known whether EBOV affects this signaling cascade, which can be beneficial or detrimental for viruses. Activation of IRE1α leads to the expression of the transcription factor XBP1s, which binds to cis-acting UPR elements (UPRE), resulting in the expression of genes aimed at restoring homeostasis in the endoplasmic reticulum. We observed that EBOV infection, in contrast to MARV infection, led to UPR activation by IRE1α-dependent but not ATF6-dependent signaling. We showed an activation of IRE1α, XBP1s and UPRE target genes upon EBOV infection. ATF6, another UPRE transcription factor, was not activated. UPRE activation was mainly attributed to the EBOV nucleoprotein NP and the soluble glycoprotein sGP. Finally, activation of UPR by thapsigargin, a potent ER-stress inducer, in parallel to infection as well as knock-out of XBP1 had no effect on EBOV growth, while MARV proliferation was affected by thapsigargin-dependent UPR activation. Taken together EBOV and MARV differ in their strategy of balancing IRE1α-dependent signaling for their own needs.

## 1. Introduction

Acute viral infections often result in an excess of newly synthesized proteins that overwhelm the protein folding ability of the infected cell. This can impose stress on the endoplasmic reticulum (ER), which leads to the activation of at least one of three signaling cascades known as the unfolded protein response (UPR) [1]. For instance, upon ER stress the UPR sensor protein PKR-like ER kinase (PERK) phosphorylates the eukaryotic translation initiation factor 2α (eIF2α) resulting in an inhibition of cellular translation. Further, the activating transcription factor 6 (ATF6) is activated by cleavage when UPR is triggered. The N-terminal part of the protein migrates into the nucleus, where it acts as a transcription factor. The most conserved UPR pathway among the three cascades, however, is initiated by Inositol-requiring enzyme 1α (IRE1α) [2]. IRE1α is activated upon ER stress resulting in autophosphorylation which triggers mRNA splicing of *X-box binding protein 1 unspliced (XBP1u)* and subsequently the translation of XBP1 spliced (XBP1s). XBP1s is transported into the nucleus to act as a transcription factor. XBP1s and active ATF6 bind cis-acting elements such as the UPR element (UPRE) which increases the expression of a plethora of genes to finally restore ER homeostasis [3,4]. Both, XBP1s and active ATF6 can bind promoter elements as homo- or heterodimer leading to the expression of different sets of genes [5,6].

Viral glycoproteins are synthesized in the ER and need to be correctly folded by cellular enzymes before they pass the quality control of the ER and are transported to the viral budding sites. The presence of too many unfolded or misfolded viral glycoproteins in the ER might overwhelm the limited capacities of the ER to properly support the folding of the proteins, creating ER stress, which in turn activates the UPR [7,8]. UPR can be beneficial or detrimental to viruses. For example, influenza A virus and adenoviruses (AdV) activate the IRE1α-dependent UPR to their advantage [9,10]. In contrast, the severe-acute respiratory syndrome-related (SARS) coronavirus (CoV) and Marburg virus (MARV) counteract the IRE1α-mediated UPR to ensure their optimal replication [11,12]. Remarkably, the potent ER-stress inducer thapsigargin (Tg), an inhibitor of the sarcoplasmic reticulum Ca2+ ATPase [13], was recently reported to counteract virus-mediated suppression of the UPR and inhibit CoV replication at non-toxic concentrations. Therefore, pharmacological manipulation of the UPR by Tg and related drugs appears as a potential strategy for the development of broad-spectrum antivirals [14,15].

Ebola virus (EBOV) and MARV belong to the family *Filoviridae* [16]. Both are notorious for causing severe illnesses in humans and are listed as priority pathogens by the World Health Organization [17]. They share similarities in their replication cycles, morphology and respective diseases [18]. In contrast, they have different strategies to counteract cellular antiviral responses such as the interferon signaling cascade [19].

The negative-stranded RNA genome of EBOV encodes seven structural proteins. The nucleoprotein NP induces inclusion bodies near the rough ER in which the polymerase L, the polymerase co-factor VP35 and the viral transcription factor VP30, together with NP, execute viral transcription and genome replication, which leads to the formation of progeny ribonucleoprotein complexes formed by the replicated genomic RNA, NP, VP35, VP24 and VP30 [20,21,22]. From there the ribonucleoprotein complexes are transported to the plasma membrane where the viral matrix protein VP40 enables budding [23,24]. Unlike MARV, which expresses only full-length glycoprotein from the GP-encoding gene [25,26], the EBOV GP gene undergoes editing by the viral polymerase to encode four main products: a membrane bound and a secreted version of the full-length GP_1,2_, the latter is the product of a proteolytic cleavage of the transmembrane anchor, a small secreted GP (ssGP) and the secreted GP (sGP), with its 5 kDa cleavage product the Δ-peptide [27]. While sGP is the primary open reading frame, stuttering of the viral polymerase at the transcriptional editing site in the GP gene leads to the insertion of one additional adenosine (A) nucleotide in the GP mRNA and the expression of the membrane-anchored full-length GP_1,2_. Co-transcriptional deletion of one A or the insertion of two A leads to the expression of ssGP [28,29]. GP_1,2_ is folded and modified in the ER and then transported via the trans-Golgi network to the plasma membrane. GP_1,2_ is post-translationally N- and O-glycosylated [30], which is important for certain functions of the protein: Single glycosylation sites within GP_2_ are essential for the intracellular transport [31] of GP_1,2_ whereas glycosylation of the mucin-like domain are needed for immune evasion [32,33]. In contrast to this, sGP is synthesized as Golgi-specific pre-sGP, which is cleaved by furin into mature sGP and the delta-peptide, which are both secreted [34,35]. While the exact function of sGP remains unclear, a role as decoy-antigen was discussed and recently, the activation of the MAP kinase signaling pathway by sGP was shown [36,37].

Recently we were able to show that the ectopic expression of MARV GP induced IRE1α-dependent signaling whereas MARV-infected cells showed no similar activation. These results could be reconciled by the finding that MARV VP30 counteracted the activation induced by GP. To ensure an efficient MARV replication a balanced UPR was beneficial [11]. As EBOV and MARV might have individual strategies to usurp host cell pathways, we wanted to elucidate if and how the IRE1α-dependent signaling is regulated by EBOV as IRE1α activation can be disadvantageous or beneficial for viral replication [9,10,11,12].

In the present study, we showed that EBOV propagation was not affected by activation of UPR by Tg treatment, while MARV propagation was reduced. In contrast to MARV infection, EBOV infection activated the IRE1α-dependent signaling cascade as shown by IRE1α-phosphorylation, XBP1s expression and UPRE activation resulting in increased target gene expression. The ectopic expression of the filoviral GPs and NPs revealed that in the case of MARV the UPRE activation was mainly mediated by GP, while for EBOV mainly sGP and NP contributed to this effect. These results support the idea that even closely related viruses, have different ways to handle cellular stress-response pathways, here the IRE1α-dependent signaling, for efficient viral replication.

## 2. Materials and Methods

### 2.1. Cell Culture

Vero C1008 (ATCC CRL-1586) and HuH7 cells were cultured as described elsewhere [11]. Vero C1008 and HuH7 cells were authenticated in 2016 by DNA profiling of eight highly polymorphic regions of short tandem repeats by the “Leibniz-Institut DSMZ (Deutsche Sammlung von Mikroorganismen und Zellkulturen) GmbH”. THP-1 cells (DSMZ no.: ACC 16) were purchased from the Leibniz-Institut DSMZ GmbH. THP-1 cells were cultured in Roswell Park Memorial Institute 1640 medium (RPMI, Thermo Fisher Scientific, Cat. No. 42401018, Waltham, MA, USA) supplemented with 10% foetal calf serum (FCS), penicillin (50 units/mL), streptomycin (50 µg/mL) (P/S) and glutamine (2 mM) (Q). To differentiate THP-1 suspension cells to adherent macrophage-like cells Phorbol 12-myristate 13-acetate (PMA, Sigma-Aldrich, P8139) was added. 1 × 10^6^ cells were seeded in 6-well plates (Corning^®^ Primaria™, Waltham, MA, USA) and stimulated with 200 nM PMA. After 48 h, the medium was replaced with fresh one and the cells were cultured for a further 5 days. Then experiments were carried out. HuH7 cells are human hepatocellular cells and THP-1 cells are macrophage-like cells. We decided to use these cells because they correspond to target cells for filoviruses [38]. HAP1 parental (Horizon Discovery, Catalog ID: C631, Waterbeach, UK) and HAP1 XBP1 knock-out (Horizon Discovery, Catalog ID: HZGHC001364c011) cells were cultured in Iscove’s Modified Dulbecco’s Medium (IMDM, Thermo Fisher Scientific, Cat. No. 12440053) supplemented with 10% FCS, P/S.

### 2.2. Virus Infection and Titration

The Mayinga strain of the species *Zaire ebolavirus* (EBOV) (GenBank accession number NC_002549) and the Musoke strain of the *Marburg Marburgvirus* (MARV) (GenBank accession number NC_001608.03) were propagated on Vero C1008 cells. The sequencing of the EBOV used revealed two amino acid mutations: eight adenosines at the transcriptional editing site of GP and a G to A substitution in the polymerase gene at nucleotide position 18138, resulting in a methionine to isoleucine replacement. Titration of the viruses was performed by plaque titration. The multiplicity of infection (MOI) was calculated based on plaque forming units per milliliter (PFU/mL) of stock viruses and is indicated in each figure.

For plaque titration Vero C1008 cells were cultured in 24-well plates to 100% confluence and infected with 10-fold serial dilutions of supernatants from infected cells. After 1 h the inoculum was removed, the cells were washed once with PBS and 2% carboxymethylcellulose (CMC, Sigma-Aldrich, C-5678, St. Louis, MO, USA) in 1× Minimum Essential Medium (MEM, Thermo Fisher Scientific, 51200-046) supplemented with 2% FCS, P/S and Q was added. At day 3 (MARV) or day 5 (EBOV) post infection (p.i.) cells were fixed with 4% paraformaldehyde (PFA) in Dulbecco’s modified Eagle’s medium (DMEM, Thermo Fisher Scientific, 21969-035) for two days. After 1 day the 4% PFA was renewed and the plates were removed from the biosafety level 4 (BSL4) facility. After the second day the cells were rinsed three times with PBS and permeabilized with PBS containing 0.1% Triton X-100 for 10 min. Thereafter, cells were washed three times with PBS and incubated with 100 mM glycine in PBS for 10 min. After a wash with PBS, the cells were incubated in blocking solution (BS, 2% bovine serum albumin, 0.2% Tween 20, 5% glycerol in PBS). The plaques were stained with the respective goat serum and secondary antibody. Plaques were counted using an Axiomat fluorescence microscope (Zeiss) and PFU/mL were calculated.

### 2.3. Antibodies

Anti-EBOV and anti-MARV sera from goat were used for the detection of VP40 and GP proteins in western blot (1:2000) and for plaque titration (1:200 in BS). Filovirus-specific goat sera were obtained after three immunizations of goats with purified gamma-irradiated virus preparations. In addition to other viral proteins, the sera have been shown to detect MARV and EBOV VP40 and GP [39,40]. Additionally, chicken-derived antibodies against EBOV NP and MARV NP (1:2,000) were used for the detection of the viral proteins by western blot. These antibodies were generated and purified in the laboratory of Prof. Schade (Charité, Berlin, Germany) as described by Pauly et al. [41]. Hens were immunized every 4–5 weeks with 0.5–1 mL of recombinantly expressed full-length EBOV or MARV NP protein. The expression and native purification of EBOV and MARV NP was carried out by GenExpress, Berlin (GenExpress is now part of TIB Molbiol). Concentrations were 0.65 mg/mL or 0.72 mg/mL NP for EBOV or MARV, respectively. For immunofluorescence analysis, a chicken-derived antibody against EBOV NP (dilution of 1:100) or a mouse monoclonal anti-MARV NP (clone 59-9-10, 1:100) were used. Hybridoma cells producing the anti-MARV NP 59-9-10 (IgG_2a_) were generated by BioGenes GmbH, Berlin, Germany. Briefly, purified gamma-irradiated MARV particles were used for four immunizations of BALB/c mice. After positive screening by ELISA, the mice were euthanized and their spleen cells fused with SP2/0 myeloma cells. After further screening and cloning twice using limiting dilution, the monoclonal cells were frozen. Endogenous IRE1α (#3294, Cell Signaling, 1:1000) and endogenous ATF6 (clone 1-7, ABIN2451924; 1:2000) were detected according to the instructions of the supplier. A mouse monoclonal antibody was used for the detection of tubulin (Clone DM 1A, 1:1000, Sigma-Aldrich), a rabbit anti-Flag antibody (1:500, Sigma-Aldrich Cat. No. F7425) was used to detect Flag-tagged XBP1 and ATF6 proteins and a rabbit anti-HA antibody was used to detect HA-tagged NPs (1:500, Rockland, Royersford, PA, USA, 600-401-384).

Secondary anti-rabbit (donkey) (Dianova, 711-036-152, Geneva, Switzerland) and anti-mouse (donkey) (Dako, P0447) antibodies conjugated to horseradish peroxidase were used at a dilution of 1: 30,000 and secondary anti-goat and anti-mouse IRDye^®^ 680 (Thermo Fisher Scientific, A21084; LI-COR, 926-68072) or anti-chicken IRDye^®^ 800 (LI-COR, 926-32218) antibodies from donkey were used at a dilution of 1: 5,000 in western blot. Secondary antibodies from goat conjugated to Alexa Fluor^®^ 594 (Thermo Fisher Scientific, Cat. No. A11042 and A11005) or Alexa Fluor^®^ 488 (Thermo Fisher Scientific, Cat. No. A11001) were used for immunofluorescence analysis and a secondary antibody from rabbit conjugated to Alexa Fluor^®^ 488 (Thermo Fisher Scientific, Cat. No. A27012) was used for plaque titration (all used at a dilution of 1:500).

### 2.4. Plasmids

The plasmids encoding the EBOV proteins sGP and GP_1,2_ [39] and the MARV proteins GP and HA-NP and the Flag-XBP1s-GFP (pCAGGS-Flag-XBP1-GFP) [11] constructs are described elsewhere. An HA-tag was cloned to the C terminus of the wild-type EBOV NP [42] by means of primer-specific PCR. The exact cloning strategy can be provided upon request. All proteins are expressed from a pCAGGS vector. The sequence analysis confirmed that the constructs are correct. The p5xUPRE-GL3 construct [43,44], which encodes the firefly luciferase controlled by a UPRE promoter was obtained from K. Mori (Department of Biophysics, Graduate School of Science, Kyoto University, Japan). A plasmid encoding the Renilla luciferase (pGL4.73, E6911) was purchased from Promega. The p3xFLAG-ATF6 plasmid was a gift from Ron Prywes (Addgene plasmid #11975) [45].

### 2.5. UPRE Luciferase Reporter Assay

Luciferase assays were performed using HuH7 cells as described by Rohde et al., 2019. Briefly, cells were transfected with 1 µg of the p5xUPRE-GL3 and 0.1 µg of the pGL4.73 construct for normalization purposes. To analyze UPRE activation by viral proteins, the cells were additionally transfected with 1 µg of the respective plasmid (MARV GP or HA-NP; EBOV NP-HA, GP_1,2_sGP) or infected with the respective virus (24 h p.t., MOI = 1). As positive control cells were treated with 300 nM or 5 nM thapsigargin for 16 to 20 h (Tg, Sigma-Aldrich, T9033). The corresponding amount of solvent DMSO was included as a negative control. Luciferase assays were performed 48 h after infection or transfection using the Beetle-Juice and Renilla-Juice BIG KITs (PJK).

### 2.6. qRT-PCR Analysis

qRT-PCR analysis was performed using Huh7 cells. Briefly, Huh7 cells were infected with MARV or EBOV with an MOI of 1. Control cells were treated with Tg (100 nM). 24 or 48 h p.i., cells were harvested, RNA was isolated and reverse transcribed using random hexamer primer and the RevertAid First Strand cDNA Synthesis Kit (Thermo Fisher Scientific, K1622). qRT-PCR analysis was performed using the Luna^®^ Universal qPCR Master Mix (NEB, #M3003L), 50 ng RNA and 250 nM Primer per reaction (Primers see Supplementary Table S1 and [46,47]). ΔCT values were normalized on the CT-values of control gene ribosomal protein S18 (RPS18), ΔΔCT-values were calculated as induction over mock-infected cells.

### 2.7. XBP1u Splicing

Analyses of the *XBP1*-mRNA variants by RT-PCR and western blot were performed as described by Rohde et al., 2019. Briefly, for western blot the pCAGGS-Flag-XBP1-GFP construct was transfected into HuH7 cells. Cells were infected 24 h after the transfection and harvested 48 h p.i. to detect the Flag-XBP1u and Flag-XBP1s-GFP protein using a Flag-tag-specific antibody. To induce XBP1 splicing, HuH7 cells were treated with 5 nM Tg for 16 h. Flag-XBP1u and Flag-XBPs-GFP signals were quantified using the Image Lab™ software and the ChemiDoc™ XRS^+^ System (BIO-RAD, Hercules, CA, USA).

For RT-PCR the cellular RNA was isolated and reverse transcribed. The cDNA was amplified using *XBP1*-specific primers that surrounded the splice site of the *XBP1* mRNA. The PCR amplificates obtained were digested with *PstI* in order to distinguish the variants of *XBP1* mRNA (only *XBP1u* mRNA, but not *XBP1s* mRNA can be digested with *PstI*). *XBP1*-mRNA variants were analyzed by 4% agarose gel electrophoresis and subsequent staining with ethidium bromide. Signal intensities of the different mRNA variants were quantified using the Odyssey^®^ CLx imaging system. The amount of *XBP1s* mRNA was set in relation to the total amount of *XBP1u* and *XBP1s* mRNA detected, the *XBP1u/XBP1s* dimer was not quantified. THP-1 cells were treated with 300 nM tunicamycin (Tun, Sigma-Aldrich Cat. No. T7765) for 24 h to induce *XBP1u* mRNA splicing.

### 2.8. ATF6 Cleavage Assay

Cleavage of ATF6 was analyzed using the plasmid p3xFlag-ATF6 as described by Rohde et al., 2019. Briefly, HuH7 cells transfected with the plasmid p3xFlag-ATF6 were infected 24 h after the transfection and harvested 48 h p.i. to detect the Flag-tagged ATF6 and its N-terminal cleavage product.

### 2.9. Western Blot Analysis

Whole-cell extracts were prepared using cell lysis buffer (Cell Signaling, #9803) as described by Krähling et al., 2009 [48]. The proteins were separated by means of SDS-PAGE and transferred to nitrocellulose membranes (Amersham Protran 0.45 NC). The membranes were incubated in phosphate-buffered saline (PBS) containing 10% skimmed milk or as recommended by the supplier of the respective antibody to block non-specific signals. Immunostaining was performed using the following antibodies in PBS containing 1% (*w*/*v*) skimmed milk and 0.1% Tween-20: anti-MARV and anti-EBOV goat serum, anti-EBOV and anti-MARV NP (chicken) and anti-Tubulin (mouse). Endogenous IRE1α and endogenous ATF6 were detected according to the manufacturer’s instructions. Western blot detection was performed using either POD-conjugated secondary antibodies and the ChemiDoc™ XRS^+^ System (BIO-RAD) or IRDye^®^ 680 or 800 secondary antibodies using the Odyssey^®^ CLx imaging system.

### 2.10. Indirect Immunofluorescence Analysis (IFA)

IFA was performed as described previously [49]. Viral nucleoproteins were stained using an antibody against MARV NP (mouse) and a chicken-derived antibody against EBOV NP both in combination with a species-specific Alexa Fluor^®^ 594-conjugated or Alexa Fluor^®^ 488-conjugated secondary antibody. DAPI (4′,6′-diamidino-2-phenylindole) staining of the nuclei was performed at a final concentration of 0.5 µg/mL. Images were acquired using a Spot inside B/W QE digital camera (Visitron Systems, Puchheim, Germany) on a Zeiss Axiophot upright fluorescence microscope (63× objective) or a LEICA DMI6000 B fluorescence microscope (63× objective, Leica Microsystems, Wetzlar, Germany) with a Leica DFC 360 FX camera (Leica Microsystems, Wetzlar, Germany).

### 2.11. Statistical Analyses

GraphPad Prism version 9.4 (GraphPad software Inc., San Diego, CA, USA) was used for statistical analysis and figure generation. Sample sizes are shown in each figure or figure legend. If applicable, each circle represents a biological replicate and comes from an independent experiment; the data are presented as the mean ± SD. Unpaired two-tailed *t* test was used to compare two data sets. Comparison among groups were done by one-way ANOVA test with Tukey’s multiple comparison post-test. The following significance levels were applied: * *p* ≤ 0.05; ** *p* ≤ 0.01; *** *p* ≤ 0.001.

## 3. Results

**Figure 1:**
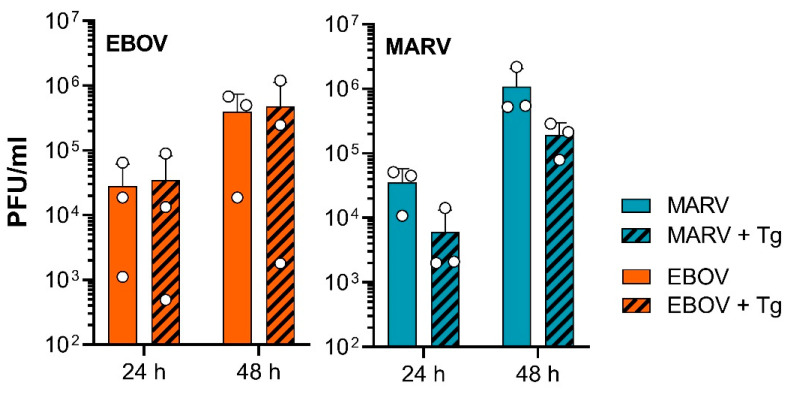
UPR activation by Tg does affect EBOV and MARV differently. HuH7 cells were infected with MARV or EBOV at a MOI of 0.01. Immediately after infection, the respective cells were treated with 5 nM Tg or with the solvent DMSO. Supernatants were collected at the indicated times after infection and analyzed for infectious virus by plaque titration.

UPR activation can be pro- or anti-viral. Recently, it was shown that Tg treatment counteracts CoV-mediated suppression of the UPR and thereby inhibits CoV replication [14,15]. Since optimal MARV replication requires tight regulation of the IRE1α-dependent signaling of the UPR [11], we wanted to analyze the effect of Tg treatment on filovirus replication. For this purpose, HuH7 cells were infected with MARV or EBOV and treated directly after infection with 5 nM Tg. Titration of infectious virus in the supernatant 24 and 48 h post infection (p.i.) showed that Tg had no effect on EBOV proliferation, while the amount of infectious MARV was reduced in Tg-treated cells at both time points (Figure 1).

**Figure 2:**
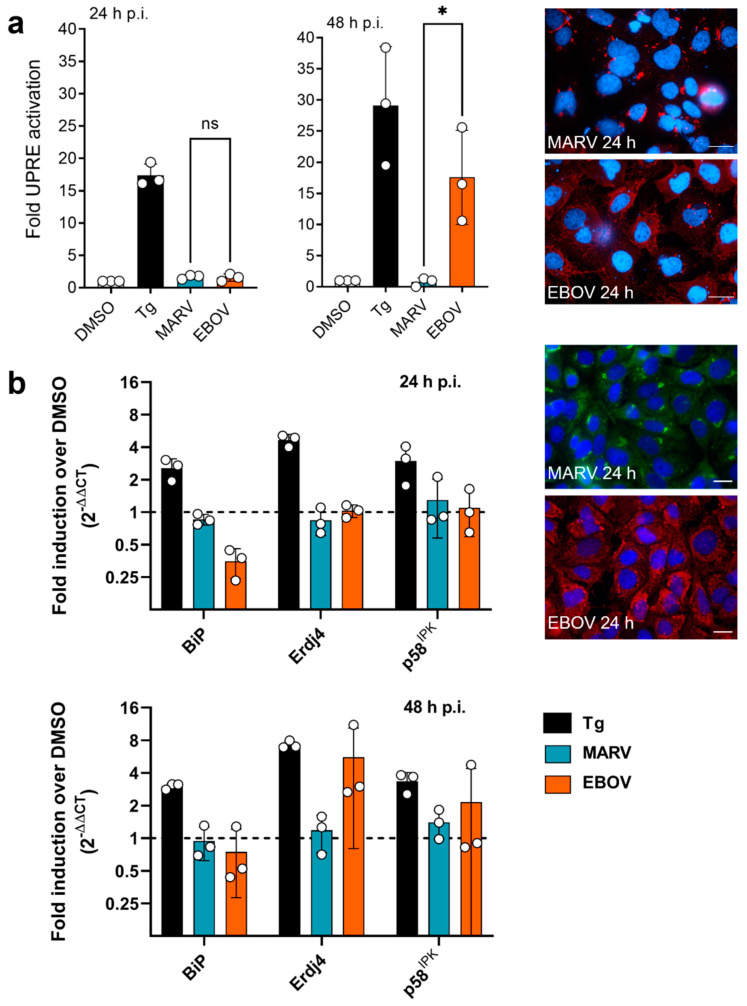
EBOV infection activates UPRE in HuH7 cells. (**a**) Cells were transfected with an UPRE-luciferase construct and with pGL4.73, which encodes Renilla luciferase for normalization purpose. Cells treated with vehicle (DMSO) or with Tg (300 nM) served as controls. 24 h after the transfection cells were infected with the respective virus (MOI = 1). The cells were analyzed using luciferase assays at the times indicated. The UPRE luciferase assay data for DMSO, Tg and MARV have already been published in Rohde et al., 2019 [11]. Since the EBOV infections were performed in the same assays, they are shown again here. From the same wells coverslips were removed just before the lysis, cells were fixed and subjected to IFA using antibodies against the NPs. Scale bar = 20 µm; Asterisks indicate statistical significance as detailed by bars between groups: * *p* ≤ 0.05. (**b**) Cells were infected with MARV or EBOV (MOI = 1). Cells treated with Tg (100 nM) or the vehicle DMSO served as control. 24 and 48 h p.i, cellular RNA was isolated. Two-step qRT-PCR analysis for the targets BiP, Erdj4 and p58IPK was performed. Data shown are normalized to the cellular control gene RPS18 and presented relative to DMSO-treated, uninfected cells. From the same wells coverslips were removed just before lysis, cells were fixed and subjected to IFA using antibodies against the NPs. Scale bar = 20 µm.

Since MARV has been shown to regulate IRE1α-dependent signaling [11], we were interested in whether the differences observed between MARV and EBOV upon Tg treatment might result from the interplay with this signaling cascade. We started by analyzing UPR activation in infected HuH7 cells using a luciferase-based reporter assay (p5xUPRE-GL3), in which expression of firefly luciferase depends on the upregulation of cis-acting UPRE by the transcription factors XBP1s and ATF6. HuH7 cells were transfected with p5xUPRE-GL3 and pGL4.73 before being infected with EBOV or MARV. Immunofluorescence analysis (IFA) showed that almost every cell was infected with both viruses 24 h p.i. (Figure 2a). The UPRE-dependent luciferase expression revealed that EBOV infection, in contrast to MARV infection, led to the activation of the UPRE at 48 h p.i (Figure 2a). We then investigated whether the observed UPRE activation led to the induction of UPR target genes in EBOV-infected cells. For this purpose, we performed qRT-PCR analysis for the following UPR target genes: *Heat Shock Protein Family A member 5* (HSPA5, *Binding Immunoglobulin Protein*, BiP), *endoplasmic reticulum DNA J domain-containing protein 4* (Erdj4) and *DNAJ homolog subfamily C member 3* (p58^IPK^). While BiP mRNA expression is regulated by ATF6 and XBP1s, Erdj4 and p58IPK are target genes of XBP1s and thus of the IRE1α-dependent UPR signaling pathway [4,50]. HuH7 cells were infected with MARV or EBOV and cellular RNA was isolated 24 or 48 h p.i. Cells treated with Tg served as a positive control. qRT-PCRs were performed and the fold induction over DMSO-treated and uninfected control cells is shown in Figure 2b. Analyzes showed that 24 h after infection with MARV and EBOV, the UPR target genes Erdj4 and p58^IPK^ were not regulated compared to control cells, whereas BiP mRNA levels were downregulated after infection with EBOV, but not MARV. IFA showed that almost every cell was infected after just 24 h (Figure 2b). After 48 h, mRNA levels of UPR target genes were still unregulated in MARV-infected cells, whereas for EBOV Erdj4 and partially p58^IPK^ were induced. BiP mRNA levels in EBOV-infected cells were comparable to those of control cells (Figure 2b).

**Figure 3:**
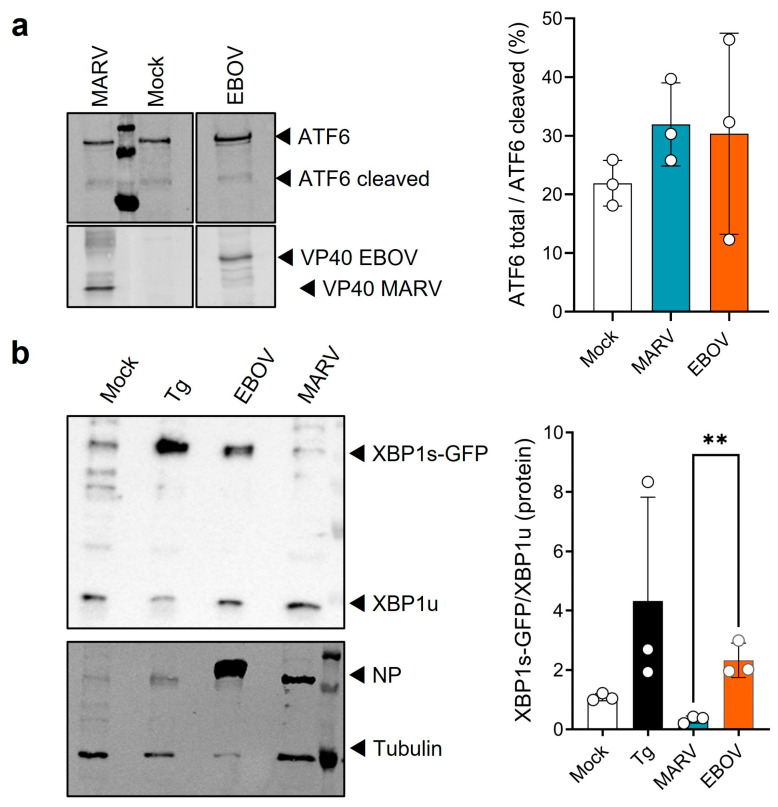
EBOV infection activates XBP1s in HuH7 cells. (**a**) Cells transfected with a Flag-ATF6 plasmid (1 µg) were infected after 24 h p.t. (MOI = 1). Cell lysates were analyzed by western blot using an anti-Flag antibody to detect ATF6 and a virus-specific goat serum to detect viral proteins. Quantification was performed using an Odyssey imaging system. (**b**) Cells transfected with a plasmid encoding Flag-XBP1s-GFP (1 µg) were infected after 24 h p.t. (MOI = 1). *XBP1u* splicing was induced by 5 nM Tg for 24 h. The cells were lysed at 48 h p.i. and analyzed by western blot using antibodies against the Flag-tag, tubulin and the nucleoproteins of both viruses. XBP1 proteins (XBP1s and XBP1u) were quantified using the ChemiDoc imaging system. Asterisks indicate statistical significance as detailed by bars between groups: ** *p* ≤ 0.01.

UPRE-dependent expression can be induced by XBP1s and ATF6 homo- and heterodimers [5]. Therefore, both proteins were analyzed by western blot to understand which of the corresponding signaling cascades are regulated during EBOV infection. HuH7 cells were transfected with plasmids coding for Flag-ATF6 (p3xFLAG-ATF6) and Flag-XBP1-GFP (pCAGGS-Flag-XBP1-GFP), respectively. Afterwards the cells were infected with either MARV or EBOV and 48 h p.i. the cell lysates were analyzed. Neither MARV nor EBOV infection led to an increased cleavage and thus activation of ATF6 compared to the mock control (Figure 3a).

The activation of XBP1s was investigated by detecting the XBP1s-GFP and XBP1u proteins. The signals were quantified and the ratio of XBP1s-GFP to XBP1u calculated. The control treatment of HuH7 cells with Tg increased the XBP1s-GFP/XBP1u ratio compared to the DMSO control (Figure 3b). Infection of the cells with EBOV also led to an increase in this ratio. This was significantly different from MARV infection.

**Figure 4:**
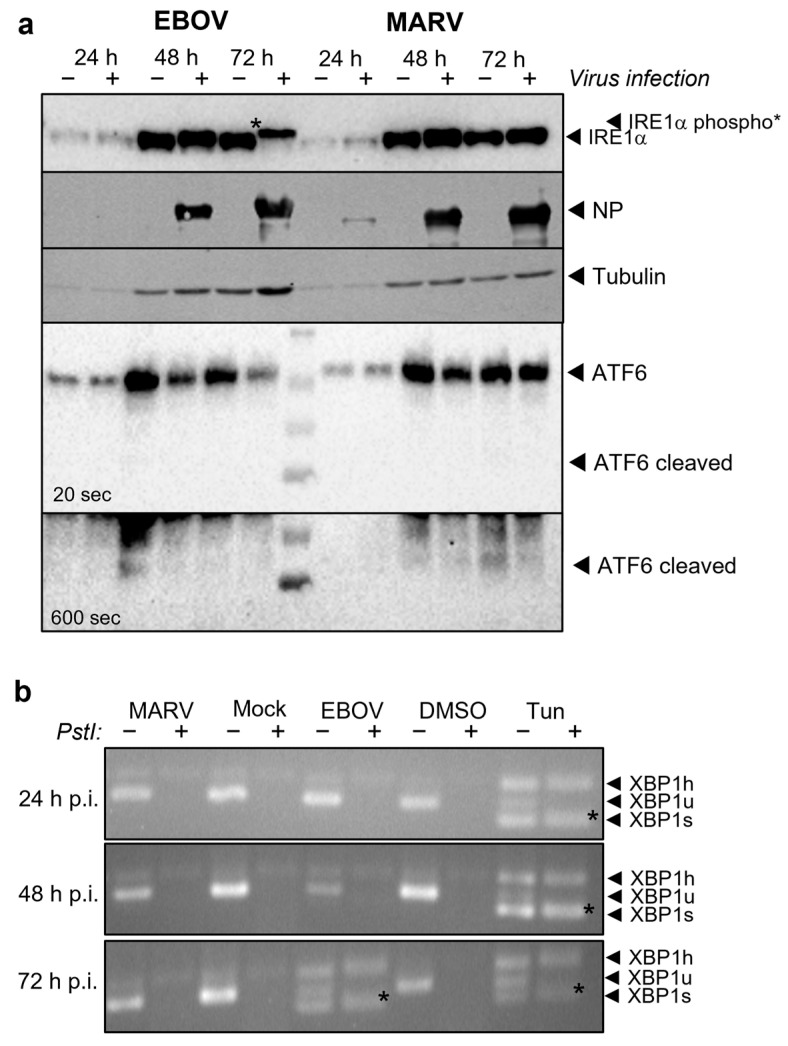
EBOV infection activates IRE1α-dependent XBP1 splicing in THP-1 cells. (**a**) Differentiated THP-1 cells were infected at a MOI of 0.1. Cells were lysed at the times indicated and endogenous IRE1α, ATF6, tubulin and the viral NPs were detected. Different exposure times are shown for ATF6 staining. The experiment was carried out three times, a representative experiment is shown. +/− indicate whether the cells were infected (+) or not (−) (**b**) XBP1-specific RT-PCR of RNA derived from cells infected as described in (**a**). Cells treated for 24 h with vehicle (DMSO) or with Tun (300 nM) served as controls. The experiment was carried out two times, a representative experiment is shown. Spliced *XBP1* mRNA (XBP1s, marked with an asterisk *) was only detected in cells treated with Tun or infected with EBOV for 72 h. +/− indicate whether PCR products were digested with *PstI* (+) or not (−). As published by others [52] we detect that XBP1u and XBP1s form a hybrid (XBP1h, confirmed by sequencing) that is resistant to *PstI* digestion.

Macrophages are primary target cells of MARV and EBOV infection [51]. To analyze whether filovirus infection also influence the IRE1α-dependent *XBP1u* splicing in macrophages, the macrophage-like THP-1 cell line was infected with MARV and EBOV. IFA showed the progression of the infection over time. After 72 h p.i., almost all THP-1 cells were infected with the respective virus (Figure S1). Western blot analysis showed that only EBOV, but not MARV infection, resulted in a clear phosphorylation of endogenous IRE1α 72 h p.i. (Figure 4a). Phosphorylated IRE1α specifically splices the *XBP1u* mRNA, resulting in the expression of XBP1s. *XBP1*-specific RT-PCR can be used to differentiate the different *XBP1*-specific mRNAs in cells (Figure S2). Tunicamycin (Tun), a substance which blocks N-linked glycosylation and thus induces accumulation of glycoproteins in the ER, served as a positive control to induce the splicing of the *XBP1u* mRNA [11]. Analysis of the RNA from MARV- or EBOV-infected THP-1 cells showed that only EBOV infection induced splicing of the *XBP1u* mRNA 72 h p.i. (Figure 4b). Furthermore, using western blot analysis, we were able to show that endogenous ATF6 is not cleaved in THP-1 cells during MARV or EBOV infection (Figure 4a). These results confirm the results gained with HuH7 cells.

**Figure 5:**
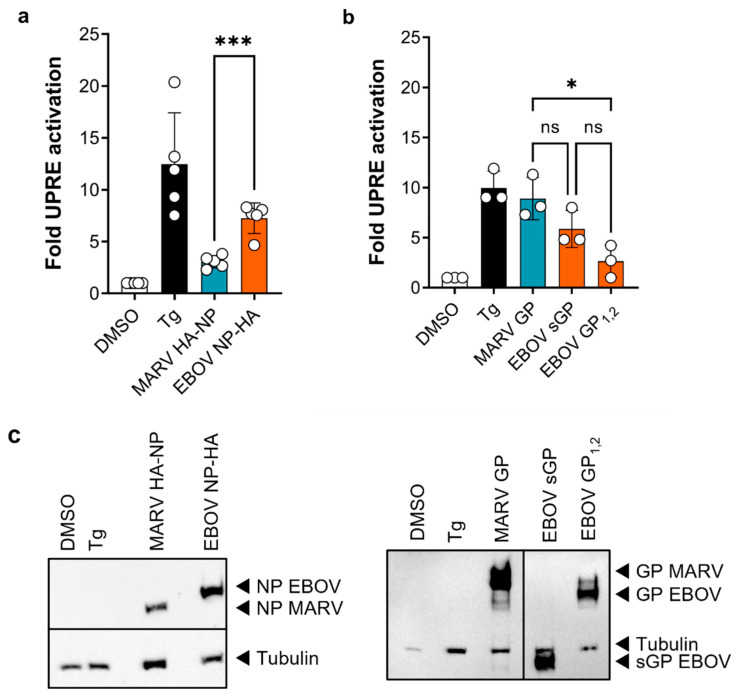
EBOV NP and EBOV sGP activate the UPRE reporter in HuH7 cells. (**a**) Cells were transfected with the UPRE reporter plasmid, with pGL4.73, and with plasmids encoding EBOV NP, MARV NP or with empty vector (DMSO and Tg). 48 h after transfection (p.t.), luciferase activities were determined. (**b**) Cells were transfected with the UPRE reporter plasmid, with pGL4.73, and with plasmids encoding MARV GP, EBOV sGP or GP_1,2_ or with empty vector (DMSO and Tg). 48 h p.t. luciferase activities were determined. Asterisks indicate statistical significance as detailed by bars between groups: * *p* ≤ 0.05; *** *p* ≤ 0.001. (**c**) Expression of the indicated proteins in the samples from (**a**) (**left**) and (**b**) (**right**) was confirmed by western blot using antibodies against tubulin and the HA-tag (**left**) or by virus-specific goat serum (**right**).

Since we observed activation of the IRE1α-dependent signaling cascade during EBOV infection, which led to activation of the UPRE-promotor, we wanted to further investigate which viral proteins are responsible for UPRE activation. Filoviral GPs are produced, folded, and modified in the ER while the NPs form inclusion bodies next to the ER [21,23,53]. Therefore, both proteins could possibly trigger the UPR. To determine whether GP or NP are responsible for UPRE activation detected during EBOV infection, UPRE-based luciferase assays were performed. HuH7 cells were transfected with p5xUPRE-GL3, pGL4.73 and plasmids encoding EBOV NP-HA or MARV HA-NP. These tests showed that EBOV NP-HA, in contrast to MARV HA-NP, strongly activates UPRE reporter activity (Figure 5a). Western blot analysis using HA-tag specific antibody showed increased NP protein expression for EBOV compared to MARV (Figure 5c). Since we had previously shown that ectopic expression of MARV GP resulted in UPRE reporter activity [11], we also investigated this for EBOV GP. The EBOV GP gene encodes four products, the most common being the sGP (about 80%) and the full-length GP_1,2_ (about 20%) [28]. To assess the influence of the two most abundant EBOV GP proteins on UPR, UPRE-based luciferase assays were performed upon ectopic expression by the respective expression constructs. MARV GP served as positive control. We observed that, in addition to MARV GP, EBOV sGP also led to a strong activation of the UPRE reporter (Figure 5b). Interestingly, the full-length glycoproteins: MARV GP and EBOV GP_1,2_ behaved significantly differently. Western blot analysis confirmed the expression of MARV GP and EBOV sGP and GP_1,2_, with no significant differences between the signal intensities of the EBOV GPs (Figure 5c). The expression levels of MARV GP and EBOV GP cannot be compared because different sera were used for detection.

**Figure 6:**
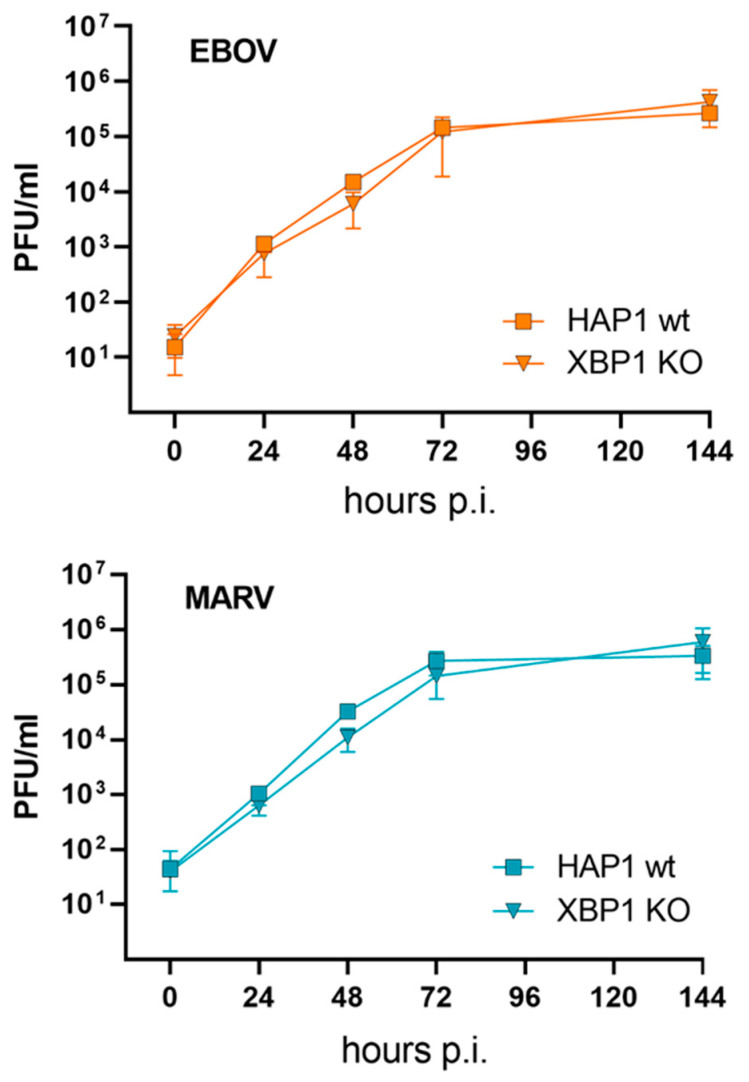
XBP1 KO does not affect filovirus propagation. HAP1 wildtype (wt) or KO cell were infected with MARV or EBOV (MOI = 1). At the indicated time points, cell supernatant was collected. Viral titers were determined using plaque-assay. N = 4.

XBP1s is activated by EBOV infection and is a very potent transcription factor involved in the regulation of many target genes [54]. Therefore, we wanted to analyze whether EBOV benefits from activation of XBP1s. For this we used commercially available HAP1 XBP1 knockout cells (KO) and HAP1 wildtype (wt) cells and infected them with EBOV or MARV. Analysis of supernatants from 0 h to 144 h p.i. using plaque titration revealed that the growth of EBOV and MARV was not affected by the XBP1 KO (Figure 6).

## 4. Discussion

Once viruses have entered the target cell, they manipulate host cell pathways for their own benefit. As a countermeasure the host cell activates several signaling cascades to repel the intruder. These processes lead to complex interactions between viruses and the host cells. One of the most important signaling cascades with the potential to influence viral multiplication is the UPR, as it not only regulates the amount and quality of viral proteins available for the production of progeny viruses but is also involved in innate immunity [55,56]. It has been shown that all three distinct UPR-associated signal cascades can be activated due to ER stress induced by viral infection [9,10,11,12,15,57,58]. Various viral proteins are involved in these processes. The highly conserved IRE1α-dependent UPR is particularly interesting because it has been shown to influence many cellular pathways such as the regulated cell death, autophagy and cytoskeleton dynamics [59,60,61].

In addition, IRE1α-dependent signaling was shown to be important in the life cycle of some viruses, including human AdV [10], human and murine cytomegalovirus [62,63] and also MARV [11].

Since the potent ER-stress inducer Tg [64], was reported to counteract coronavirus-mediated suppression of the UPR and thereby inhibit CoV replication, the pharmacological manipulation of the UPR by Tg and related drugs appears as a potential antiviral strategy [14,15]. Furthermore, we have shown that MARV actively counteracts IRE1α activation [11], suggesting that Tg may also affect MARV replication. Indeed, we could show that Tg treatment reduced MARV proliferation, while EBOV proliferation was unaffected (Figure 1), illustrating the difference between the two viruses. Similarly, previous analyses by others showed that Tg stimulation can be pro- or anti-viral, suggesting different viruses having different sensitivities to UPR. For example, an antiviral effect due to the stimulation of UPR by Tg was found for several CoVs [14,15,65], the influenza A virus [65] and several members of the virus order *Mononegavirales*, such as the respiratory syncytial virus [65,66]. On the other hand, Tg treatment is very well tolerated by AdV and herpes simplex virus, in some cases even positively affecting viral replication [67,68].

Further, characterization of the differences between MARV and EBOV revealed that both viruses did not activate ATF6-dependent signaling and interacted differently with IRE1α-dependent signaling (Figure 2 and Figure 3). In contrast to MARV, EBOV infection induced UPRE and UPR target gene activation such as Erdj4 at 48 h p.i. (Figure 2). In contrast, BiP mRNA levels, which are predominantly regulated by heterodimers of ATF6 and XBP1s [6], were downregulated in the first 24 h of EBOV infection and upregulated within the following 24 h. Because ATF6 is not activated by EBOV, this effect could be explained by the finding that XBP1s homodimers were upregulated by EBOV 48 h p.i. which subsequently induced BiP expression. Others have shown that BiP is an essential host factor for EBOV [69] and that BiP mRNA levels were upregulated during EBOV infection of monocytes from rhesus macaques [70]. This is in contrast to our results, which show downregulation of BiP mRNA early during infection. Differences between these studies and ours are, on the one hand, that different cells from different species were used and that the infection studies described here were performed with an EBOV that carries eight adenosines (8A) instead of seven (7A) at the transcriptional editing site of the EBOV GP gene. It has been shown that authentic EBOV with 7A phenotype produces roughly 80% sGP and 20% GP_1,2_, whereas an 8A EBOV produces mainly GP_1,2_ (appr. 75%) and less sGP (appr. 10%) [71,72]. Both could be an explanation for the observed differences in BiP mRNA levels. Further studies are planned to elucidate the individual contribution of the different EBOV GP proteins.

To examine cell-type specific differences, we wanted to confirm the results from HuH7 cells using THP-1 cells. THP-1 cells are a macrophage-like cell line resembling the primary target cells of filoviruses in humans [73]. As in HuH7 cells EBOV infection activated the IRE1α-dependent signaling cascade resulting in *XBP1u* splicing, but not ATF6 cleavage (Figure 4). MARV infection of THP-1 cells did not lead to splicing of *XBP1u*, consistent with the results from infected HuH7 cells in the present study (Figure 3) and previous results [11]. Previous results showing transient IRE1α phosphorylation after MARV infection [11] could not be confirmed in THP-1 cells. This could be because they are less susceptible to filovirus infection and show a slower progression of infection than HuH7 cells (Supplementary Figure S1), in which almost 100% of the cells can be initially infected (Figure 2). In EBOV-infected THP-1 cells compared to uninfected cells, we observed that the levels of the endogenous proteins IRE1α and ATF6 decrease over time, while tubulin levels increase (Figure 4) suggesting a role for XBP1s-induced ER-associated degradation (ERAD) [54].

Since several studies showed that the expression of viral glycoproteins [7,8] such as the MARV GP [11] can lead to UPR activation, we analyzed whether the expression of EBOV GP is responsible for the activation of the *XBP1u* splicing. Surprisingly, mainly EBOV NP and sGP activated UPRE-dependent luciferase expression in HuH7 cells (Figure 5). EBOV NP is found mainly in inclusion bodies adjacent to the ER but not inside the ER [20,21]. Therefore, a direct activation from inside the ER is not likely. Since it has been shown that splicing of *XBP1u* mRNA by IRE1α can also take place without ER stress [74], activation via a cytoplasmic protein such as EBOV NP is nevertheless conceivable. IRE1α is a multifunctional protein and interacts with a variety of proteins to regulate its function under physiological and stressful conditions [75]. Whether a transient activation of IRE1α can be enhanced by direct or indirect interaction with EBOV NP has to be clarified by further experiments. Recently, others reported a UPR-activating effect of ectopically expressed EBOV GP_1,2_, leading to targeted degradation of EBOV GP_1,2_ by protein disulfide isomerases via ERAD and subsequent lysosomal degradation. Whether sGP activates UPR was not analyzed in this study, but it was not affected by protein degradation [76]. EBOV GP_1,2_ degradation via ERAD and lysosomal degradation was confirmed by others as well [77]. Both studies suggest that EBOV hijacks proteostasis pathways to downregulate EBOV GP_1,2_ expression to increase viral fitness. This selective downregulation of EBOV GP_1,2_ might explain why we observed that mainly sGP activated the UPRE. Considering the data obtained during ectopic expression of viral proteins (Figure 5) and the fact that the infection studies described here were performed with an EBOV carrying the GP 8A gene in its genome, which leads to low sGP (appr. 10% instead of 80% by 7A EBOV) and high GP_1,2_ expression (appr. 75% instead of 20% by 7A EBOV) [71,72], activation of the IRE1α-dependent signaling cascade upon infection appears to be primarily induced by NP. Further studies with recombinant EBOVs with a 7A and 8A phenotype will clarify the importance of a balanced expression of GP_1,2_ and sGP with regard to UPR activation and degradation processes as the effect of sGP might be underestimated here.

MARV and EBOV interact differently with some signaling cascades such as the oxidative stress response [78,79] and the interferon system [19], supporting the idea that both viruses, although closely related, use different strategies to deal with cellular antiviral mechanisms. Since MARV and EBOV differ in their responsiveness to Tg (Figure 1) and in their ability to activate the transcription factor XBP1s (Figure 3 and Figure 4) we assessed whether XBP1 KO affects viral growth. What we observed was that the propagation of MARV and EBOV was neither impaired nor increased in commercially available HAP1 XBP1 KO cells, suggesting that XBP1s activation does not have a critical impact on EBOV proliferation and that the difference of MARV and EBOV cannot be explained by XBP1s activation in this experimental setting. For MARV, the results are consistent with previously published data showing that MARV VP30 antagonizes IRE1α-dependent *XBP1u* splicing. Therefore, KO of XBP1s had no effect on MARV replication, but stimulation by Tg did (Figure 1). Whether the effect of Tg on MARV results from XBP1s activation or from other effects of Tg needs further investigation.

In summary, we have shown that EBOV infection leads to activation of the IRE1α-dependent signaling pathway, activation of XBP1s and thus induction of the UPRE and related target genes. UPRE activation was mainly attributed to the ectopic expression of EBOV NP and sGP. Finally, activation of the UPR by Tg as well as KO of XBP1 had no effect on EBOV growth, while MARV proliferation was negatively affected by Tg-dependent UPR activation, highlighting the differences between both viruses.

## Data Availability

Further information on the presented data as well as the raw data is available on request.

## Supplementary Materials

The following are available online at https://www.mdpi.com/article/10.3390/v15010122/s1, Supplementary Table S1: Oligonucleotides used for qRT-PCR; Figure S1: Filovirus infection of THP-1 cells; Figure S2: XBP1-specific RT-PCR [52].
