# Supplementary material for: Ebola Virus Activates IRE1α-Dependent XBP1u Splicing
Source: Viruses. 2022 Dec 30;15(1):122. doi: 10.3390/v15010122 (PMC9863596; doi:10.3390/v15010122)
Supplement: Supplementary file 1 [file viruses-15-00122-s001.zip › viruses-2096135-supplementary.pdf]

Name	Sequence (5‘-3‘)
Human BiP fwd.	CATGGTTCTCACTAAAATGAAAGG
Human BiP rev.	GCTGGTACAGTAACAACTG
Human Erdj4 fwd.	AAAATAAGAGCCCGGATGCT
Human Erdj4 rev.	CGCTTCTTGGATCCAGTGTT
Human $p58^{IPK}$ *fwd.*	CTCAGTTTCATGCTGCCGTA
Human $p58^{IPK}$ *rev.*	TTGCTGCAGTGAAGTCCATC
Human RPS18 fwd.	GCGGCGGAAAATAGCCTTTG
Human RPS 18 rev.	GATCACACGTTCCACCTCATC

Supplementary Table S1. Oligonucleotides used for qRT-PCR
fwd. indicates a forward primer, *rev.* indicates a reverse primer

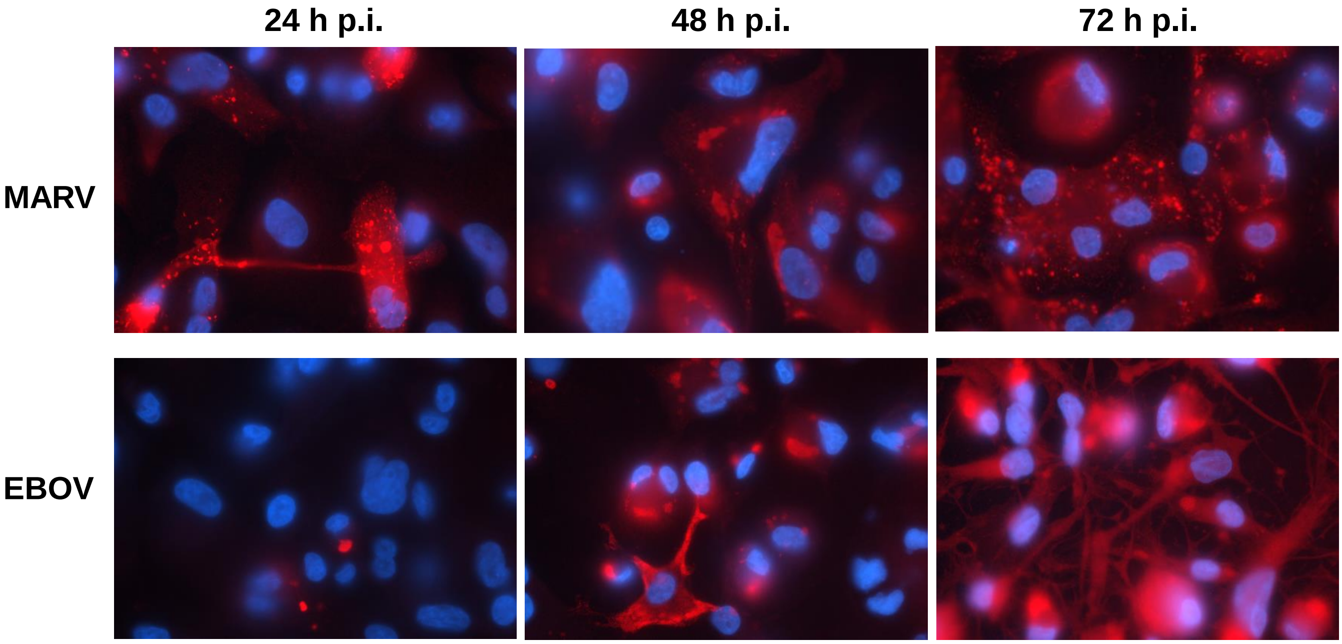

Figure S1. Filovirus infection of THP-1 cells. Cells were infected with MARV or EBOV (MOI = 0.1). The cells were fixed with 4% paraformaldehyde at the indicated time points and subjected to IFA using antibodies against the respective NP. The photomicrographs were obtained using the same exposure times. DAPI staining labels cell nuclei.

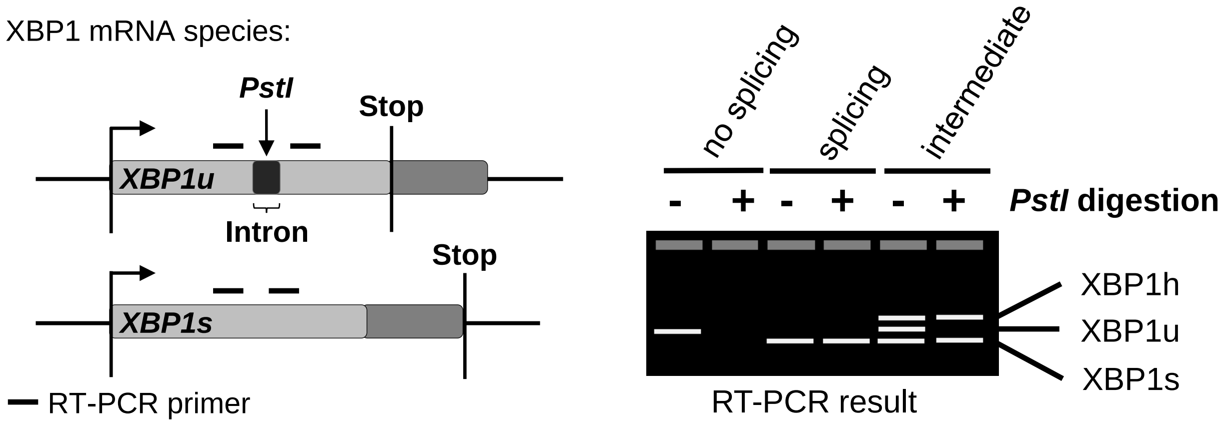

Figure S2. XBP1-specific RT-PCR. In the absence of IRE1α activity, *XBP1u* mRNA will not be spliced; The *PstI* restriction site is present and the PCR product can be digested. If IRE1α is active, *XBP1u* is spliced; The *PstI* restriction site is thereby removed and the PCR product cannot be digested. Intermediate phenotype: *XBP1u* is partially spliced and a hybrid of XBP1u and XBP1s forms (XBP1h, confirmed by sequencing) that is visible in the agarose gel and resistant to digestion. This has also been shown by e.g. Li *et.al.,* 2010 [52].
